# Using Theory of Planned Behavior to Predict the Physical Activity of Children: Probing Gender Differences

**DOI:** 10.1155/2015/536904

**Published:** 2015-11-16

**Authors:** Lijuan Wang, Lin Wang

**Affiliations:** Shanghai University of Sport, Shanghai 200441, China

## Abstract

*Objectives*. The primary objective of this study was to use the theory of planned behavior (TPB) to examine the association between TPB variables and the moderate-to-vigorous physical activity (MVPA) of children in Shanghai, China. Gender differences were also explored.* Methods*. The participants were 353 children (180 boys and 173 girls) aged 9 to 13 years from three primary schools in Shanghai. Accelerometers were used to measure the MVPA duration of the children. Questionnaires that focused on attitude, subjective norms, and perceived behavioral control (PBC) related to MVPA engagement were completed by the participants.* Results*. Regression analyses revealed that intention, and not PBC, accounted for 9% of the variance in MVPA. Meanwhile, attitude and PBC explained 33% of the variance in intentions to engage in MVPA. In terms of gender differences, TPB performed better in the physical activity (PA) domain for boys than for girls. Furthermore, attitude and PBC were significantly associated with intention among boys, whereas only PBC was significantly related to intention among girls.* Conclusion*. Practitioners should consider tailoring intervention to address gender differences to increase leisure-time PA participation of children.

## 1. Introduction

Most experts agree that physical activity (PA) benefits both physical [1, 2] and psychological health [3]. Childhood is a critical period for promoting PA because developing such activities early in life may continue into adulthood [4, 5]. Despite the benefits of PA, substantial evidence indicates that the PA of children and adolescents has declined drastically over the years in numerous countries, including the United States (US), Australia, and Canada [6–8].

In China, practices related to health and nutrition rapidly changed because of political and social reforms, which resulted in an obesity epidemic among children and adolescents [9]. The number of obese children and adolescents aged 7 to 22 years increased from 1% and 2% for boys and girls, respectively, in 1985 to approximately 7% and 5% in 1995, and 13% and 6% in 2010 [9, 10]. The low level of PA among children and adolescents is a major factor that contributes to obesity [11]. The China Health and Nutrition Survey, which was conducted among nearly 2700 youths aged 6 to 18 years from 8 provinces in China, showed that 72% of Chinese youth engaged in in-school moderate-to-vigorous PA (MVPA) for 90 to 110 minutes/week, but only 8% engaged in MVPA outside of school [12]. Wang et al. (2013) measured the MVPA of 2163 students in the 4th to 11th grade from 11 cities in China and found that Chinese city children and adolescents spent an average of 28.26 minutes/day engaged in MVPA and 521.50 minutes/day engaged in sedentary behavior [13]. All these studies showed that only a few children and adolescents met the recommended 60 minutes/day of MVPA [2]. To develop effective strategies in promoting PA, psychologists have directed their attention to psychological factors and have indicated that these factors should be identified [14, 15].

Several psychological models have been used over the past decades to improve the understanding of the antecedents of engaging in PA behavior, such as social cognitive theory, self-determination theory, and theory of planned behavior (TPB). Among these, TPB is a prominent model that has been proven to be effective, parsimonious, and versatile for examining the antecedents of PA behavior among children and adolescents [16]. According to TPB, behavioral intention is influenced by attitude, subjective norms, and perceived behavioral control (PBC) [17, 18]. In the present study, attitude represents the positive or negative evaluation of engagement in MVPA, whereas subjective norms reflect perceived social pressures to perform MVPA. PBC refers to resources and obstacles that facilitate or impede engagement in MVPA behavior [17, 18]. Meanwhile, behavior is believed to be determined by intention and PBC. Based on this study, children who have high motivation to engage in MVPA are more likely to have high MVPA levels. Second, children who express strong feelings of control over their PA are likely to be engaged in more MVPA.

TPB has been applied to examine the PA of children and adolescents from North American and European countries [15, 19–28]. Most of these studies have found that TPB variables accounted for less than 50% of the variance in intention and less than 10% of the variance in behavior. For example, Martin et al. (2007) found that TPB variables accounted for 8% to 9% of the variance in MVPA among Mexican-American children. Intention is one of the major predictors of leisure-time MVPA. Trost et al. (2002) focused on sixth-grade students in the US and noted that attitude and subjective norms are significantly related to intention and accounted for 13% of the variance in intention. PBC and intention accounted for only over 6% of the variance in MVPA. Sas-Nowosielski (2006) examined the usefulness of TPB in explaining the leisure-time PA of Polish adolescents aged 13 to 19 years. The results indicated that intention was a significant variable significantly associated with participation in leisure-time PA, and attitude and PBC accounted for 29% of intention variance. Mummery et al. (2000) also investigated the efficacy of TPB in explaining the PA intention of Canadian children and adolescents. The results showed that three TPB constructs explained 47% of the variability in measuring PA intention.

Gender is the most studied variable in PA pattern differences. Overwhelming evidence indicates that girls are less active than boys at all ages [12, 13, 29, 30]_._ Studies have also investigated gender differences in TPB variables in relation to the PA of children and adolescents [23, 24]. Boys demonstrated a more positive attitude and higher intention to engage in MVPA in the study by Martin et al. (2007). Furthermore, gender significantly influenced MVPA but not intention. In Martin et al. (2008), boys were reported to have stronger barrier self-efficacy, whereas girls had stronger perceptions of subjective norms. In addition, studies have been conducted to examine differences in explaining the TPB model for PA of boys and girls [19, 27, 28]. The results of previous research are conflicting. Beville et al. (2014) focused on college students and revealed that attitude, intention, self-efficacy, body mass index, and sports participation were significantly associated with leisure-time PA for girls, whereas intention was significant for boys. Sas-Nowosielski (2006) found that although boys perceived more social pressure to be physically active, girls were more influenced by social pressure. The association between PBC and intention was significant and moderately strong for boys but weak and insignificant for girls. However, Rhodes et al. (2006) reported that TPB performed equally in explaining intention and behavior toward PA among boys and girls in Canada. Collectively, these results highlighted the importance of considering the effects of gender differences in studies regarding children and adolescent PA.

Although studies have applied TPB to explain the MVPA of children and adolescents, limitations still exist. First, self-report measures of PA were adopted to determine the PA participation among children and adolescents in most of these studies. This measure may be subject to response bias and estimation error, particularly for children. Such limitation may have augmented or inflated the strength of relationships between TPB variables and behavior. More accurate and precise objective measures (e.g., pedometers and accelerometers) may be required to assess PA level [19, 25]. Second, these studies were conducted in North American and European countries. Few studies were conducted on using TPB to examine the PA of children or adolescents in China. Chinese culture (e.g., Confucianism) and policies (e.g., the One Child Policy) are different from those of Western countries, which is likely to result in different PA patterns of children. Third, given the inconsistent findings of research in terms of gender differences in TPB explanation of MVPA behavior, additional studies are required to confirm whether gender differences actually exist. To bridge these research gaps, the present study aims to realize the following research objectives: (1) to describe the MVPA duration of children and TPB constructs as well as their correlations, (2) to explore gender differences in the MVPA duration and TPB variables of children, and (3) to examine the association between TPB variables and intention to engage in PA as well as MVPA duration of children. If gender differences are found, then the association between TPB variables and intention and behavior of boys and girls will be separately explored and compared.

## 2. Methodology

### 2.1. Participants

The university ethics committee and relevant school educational authorities approved the study. Three primary schools were randomly selected from three districts in Shanghai, a city located in the eastern part of China. The principals were contacted in advance and all agreed to participate in the study. Primary schools have five grades (i.e., grades 1 to 5, with students aged 7 to 12 years). Grades 1 and 2 students (aged 7 to 9) were excluded from the study because they were too young to understand and complete the survey accurately. Two classes were randomly selected from each grade level (i.e., grades 3 to 5), and all the students in the two classes were invited to participate. Informed assent forms were distributed to all 561 students and their parents prior to data collection. A total of 483 students agreed to participate. Of the 483 participants, 27 were excluded because of accelerometer malfunction or loss, whereas 103 were excluded because their valid accelerometer data did not cover at least 2 valid weekdays and 1 valid weekend day. The final analytic sample consisted of 353 participants. The ages of the participating students ranged from 9 to 13 years (M = 11.26, SD = 0.98). The breakdown by gender was 173 (49%) girls and 180 (51%) boys. The participants comprised 101 (29%) grade 3, 122 (34%) grade 4, and 130 (37%) grade 5 students.

### 2.2. Measures

#### 2.2.1. Leisure-Time MVPA

PA was measured using the Actigraph GT3X Activity Monitor for seven days. This monitor is widely accepted as a valid and reliable tool for assessing MVPA among children and adolescents [31]. The children were asked to wear the monitor on their waist during waking hours and to remove the device before bathing, taking a shower, or swimming [32]. The sampling interval (epoch) in the present study was set at 30 seconds. After the test, the original Actigraph data files were downloaded to a personal computer using ActiLife software 6.5.2. Accelerometer data were considered valid if over 600 minutes (10 hours) of monitoring per day (excluding strings of zeros for 20 minutes or longer) was recorded over the entire monitoring period [33]. Accelerometer data were included in the final analysis if they contained at least 2 valid weekdays and 1 valid weekend day [13]. Chinese-specific cutoff points were used to determine activity level thresholds. These points were more suitable for Chinese children compared with previously developed cutoff points, which defined moderate PA as 2800 CPM to 3999 CPM and vigorous PA as 4,000 CPM and above [34]. Finally, MVPA levels were reported as the average daily time (minutes/day) spent in MVPA across all valid days. The present study only included MVPA that was performed during free time (i.e., outside of class time) because TPB was considered as a motivational model for understanding behavior [27]. Thus, all MVPA data from 8:00 a.m. to 4:00 p.m. (i.e., school time) were excluded from this study.

#### 2.2.2. Background Information

Each student provided a brief background about themselves, including their age, gender, and grade in the questionnaires.

#### 2.2.3. TPB Constructs

TPB scales have been used in PA research with similarly aged adolescents and have established acceptable reliability [21].* Intention* to engage in MVPA was assessed by the following three items: (1) “I plan to do physical activities that make me out of breath for at least three or more times during my free time next week,” (2) “I expect to do physical activities during my free time next week,” and (3) “I intend to do physical activities that make me out of breath for at least three or more times during my free time next week.” The responses were given using a scale ranging from 1 (unlikely) to 7 (likely).* Attitude* toward regularly engaging in MVPA in the subsequent week was assessed by the item: “Doing physical activities that make me out of breath at least three or more times during my free time next week is….” Three scales, namely, good–bad, exciting–boring, and fun–unpleasant, were used.* Subjective norms* were measured using a seven-point scale that ranged from 1 (strongly disagree) to 7 (strongly agree). A single item was included in this section, namely, “Most people who are important to me think I should do physical activities that make me out of breath at least three or more times during my free time next week.” The PBC scale included three items with seven-point scale responses: (1) “Whether I participate in physical activities that will make me out of breath at least three or more times during my free time next week is entirely up to me” (1 strongly disagree–7 strongly agree), (2) “Whether I do physical activities that will make me out of breath at least three or more times during my free time next week is mostly up to me” (1 false–7 true), and (3) “How much personal control do you feel you have over engaging in physical activities that will make you out of breath at least three or more times during your free time next week?” (1 no control–7 complete control).

### 2.3. Translation Procedures and Trustworthiness

The instrument was translated and validated prior to data collection. The translation and back-translation of the instrument used in the present study were conducted by two bilingual translators. The back-translated version was then compared with the original English version, and any inconsistencies and errors were highlighted. Differences were negotiated until the translators agreed with each other. The Chinese version demonstrated acceptable test-retest reliability of 0.84 for intention, 0.81 for attitude, and 0.74 for subjective norms. We obtained a lower reliability coefficient for PBC (0.67). The questionnaire was sent to five experts on PA for their suggestions. Consequently, slight modifications were made with several statements based on the comments of the experts. For example, the original phrase “moderate to vigorous” was replaced with “out of breath” to ensure that the children would understand the type of PA being investigated. In addition, “at least 30 minutes” and “physical activities that make me out of breath for at least three or more times” were included in the questionnaire to make the targeted PA behavior consistent with the* Chinese Guide to Healthy Active Living for Children and Youth *(1996).

### 2.4. Data Collection

Data collection was conducted from October to December 2014. Before data collection, the researchers introduced the objectives and methods of the study to the participants. The researchers obtained consent from both the children and their parents. The primary author and two graduate students majoring in sports pedagogy administered the survey and the test. Upon arrival at each school, the data collector distributed pencils and described the scales used in the questionnaire. The participants were given instructions and directed to complete the questionnaire. The survey could be completed for approximately 10 minutes. The questionnaires were collected immediately upon completion. In the second week, all participants were provided with accelerometers and were instructed to wear them for 7 consecutive days underneath their clothes and to fasten them to their right hipbone using an elastic belt. The accelerometers were initialized before using ActiLife software 6.5.2 to start collecting data at 0:00. The participants were asked to follow their normal daily routines during the monitoring period. Written instructions that remind them to wear the accelerometer were also given to all participants and their parents to increase compliance. The children were required to return the accelerometers after 8 days to ensure 7 days of complete data collection.

### 2.5. Data Analysis

Descriptive statistics were calculated for MVPA duration and TPB scales by adding the items on each scale and dividing the sum by the number of items on the scale. Pearson product-moment correlations were calculated to examine the interrelationships among all TPB variables. Multivariate ANOVA (MANOVA) was performed using MVPA duration and TPB variables as dependent variables and gender as independent variable. For significant MANOVAs, univariate follow-ups were conducted to determine significant differences that occurred. Eta-squared (*η* ^2^ = 1–Wilk's lambda) was used as effect sizes for multivariate effects [35]. Finally, hierarchical multiple regression analyses of intentions and behavior were conducted on the TPB variables.

## 3. Results

### 3.1. Descriptive Findings

Table 1 presents the mean scores and standard deviation. Based on the Chinese-specific cutoff points, the participants spent an average of 15.26 (SD = 11.21) minutes/day during their free time in engaging in MVPA. The mean intention score (M = 3.93, SD = 1.77) was close to the midpoint, but the scores of attitude (M = 4.99, SD = 1.76), subjective norms (M = 5.21, SD = 1.82), and PBC (M = 4.64, SD = 1.64) were higher than the midpoint.

Table 1 also presents the bivariate Pearson product-moment correlations among all variables in this study. All variables were weakly to moderately associated with one another except for the insignificant relationships between attitude and MVPA (*r* = 0.07, *p* > 0.05) as well as subjective norms and MVPA (*r* = 0.06, *p* > 0.05).

### 3.2. Gender Differences in MVPA and TPB Variables

Given the significant correlation among MVPA, attitude, subjective norms, and PBC, MANOVA was employed to examine gender differences in these aspects (Table 2). The results indicated that gender differences were significant in MVPA and TPB variables (*F*(1, 353) = 2.63, *p* < 0.05, and *η* ^2^ = 0.04). The results of the follow-up univariate test revealed that MVPA time (*F*(1, 353) = 6.59, *p* < 0.05, and *η* ^2^ = 0.02) and intention to engage in PA (*F*(1, 353) = 4.80, *p* < 0.05, and *η* ^2^ = 0.01) contributed to the significant gender difference. The MVPA of boys (M = 16.75, SD = 12.53) was significantly higher than that of girls (M = 13.71, SD = 9.43). Moreover, boys expressed significantly higher intention (M = 4.14, SD = 1.84) to participate in PA than girls (M = 3.72, SD = 1.69). However, no significant difference was observed in attitude, subjective norms, and PBC between boys and girls.

### 3.3. Regression Analysis of Explaining Behavior and Intention

Multiple regression analyses were conducted to examine the association between TPB variable and MVPA duration of children. We first entered intention because TPB postulated that intention was the primary variables associated with behavior. In the second block, we entered PBC because it was believed to influence behavior directly apart from affecting behavior through intention. In the third block, we entered the remaining TPB constructs (i.e., attitude and subjective norms) because they were hypothesized to only influence intention directly with no direct path to behavior (i.e., MVPA). Lastly, considering the previously reported gender differences, we entered gender to determine whether this factor would be associated with MVPA time.  Table 3 presents the results of this analysis. In the first three stages, all TPB variables explained 9% of the variance in the MVPA of children (*F*(3, 353) = 9.644, *p* < 0.001). Intention was the only variable significantly related to MVPA. The addition of gender in Step  3 accounted for 1% more of the variance in behavior (*F*(4, 353) = 8.727, *p* < 0.001). Intention and gender were significantly related to behavior.

To examine the association between three TPB variables and the intention of children to engage in PA, all TPB variables were entered simultaneously in the first step and gender was entered in the second step. The results for the analysis are presented in Table 4. Step  1 variables accounted for 33% of the variance in intention (*F*(1, 353) = 59.178, *p* < 0.001), with attitude and PBC reported as significant. Step  2 variables significantly accounted for an additional 1% in intention variance (*F*(2, 353) = 47.208, *p* < 0.001). Attitude, PBC, and gender were significantly related to intention.

### 3.4. Gender Differences in Explaining Behavior and Intention

Given that gender was a significant factor related to intention and behavior among children, separate regression models were analyzed to examine gender differences in the TPB structure. The results shown in Table 5 revealed that TPB variables explained 10% of the variance in MVPA among boys (*F*(3, 180) = 5.849, *p* < 0.001). In addition, the standardized beta weights suggested that intention was the sole variable significantly associated with MVPA. With regard to girls, the results showed that all TPB variables accounted for 6% of the variance in MVPA (*F*(3, 173) = 3.783, *p* < 0.01), with intention as the sole factor that is significantly related to MVPA. In terms of intention, Table 6 indicates that TPB variables explained 39% of the variance in behavioral intention among boys (*F*(1, 180) = 39.445, *p* < 0.001). The standardized beta weights suggested that PBC, followed by attitude, was the most important variable. Meanwhile, TPB variables explained 29% of the variance in behavioral intention among girls (*F*(1, 173) = 24.705, *p* < 0.001). Unlike that among boys, only PBC emerged as a significant variable related to MVPA among girls.

## 4. Discussion

### 4.1. Descriptive Findings

The moderate and high mean levels of all TPB variables reported by the children who participated in this study were encouraging because they reflected a positive view toward MVPA. The respondents were neutral in their intentions to engage in MVPA, but they expressed favorable attitude toward MVPA and were motivated to comply with the wishes of others that they should be active. Lastly, they reported a strong sense of control over their ability to engage in MVPA if they chose to. This favorable cognition of MVPA among Chinese children is similar to those in previous studies [23–25]. In terms of MVPA among children, the respondents spent an average of 15.26 minutes/day during their free time engaged in MVPA. Although the World Health Organization (2010) recommended at least 60 minutes of MVPA per day among children and adolescents, the organization offered no recommendation for specified leisure-time PA. Therefore, we cannot determine whether the children included in the present study are physically active or inactive during free time. However, compared with other studies, the leisure-time MVPA duration of children in this work was shorter [8, 23, 36–38]. In Pahkala et al. (2007), heart rate monitors were used to obtain data on MVPA of 13-year-old Finnish girls outside of school. The findings showed that sedentary, moderately active, and active girls spent an average of 28, 31, and 33 minutes daily, respectively, in MVPA. The corresponding durations for boys were 33, 47, and 38 minutes. Klinker et al. (2014) used accelerometers to measure the PA of children from Denmark outside of school with a mean age of 12 years and found that they spent an average of 18 minutes engaged in leisure-time MVPA each day. Shores and West (2010) quantified leisure-time PA among college students in the US using a short questionnaire and a 2-day time diary. The respondents in this study reported 36 minutes of PA during leisure time and 56 minutes during weekends. Martin et al. (2008) indicated that Arab-American children aged 10 to 14 years spent slightly over 2 hours engaged in MVPA during their outside-school time each week.

### 4.2. Testing TPB in the PA Domain

The results of this study provided support for TPB by demonstrating that attitude and PBC were significantly associated with intentions to engage in MVPA among children and that intention, not PBC, emerged to be significantly related to MVPA among children after a 1-week follow-up period. The findings revealed that 9% of MVPA and 33% of intention were explained by TPB variables in the present study. This finding is similar to those of several previous studies [15, 20, 24–26, 28], which found that TPB accounted for 4% to 9% of the variance in MVPA among children and less than 50% of the variance in their intention. Based on this finding, TPB was not a particularly useful explanatory framework for MVPA variance because 67% of the variance in intention and 91% of the variance in behavior could not be explained by TPB. Similarly, several researchers reported that the TPB is limited because it cannot provide an acceptable explanation for human behavior and that it needs to be changed or extended [16, 39, 40]. Moreover, Sniehotta et al. (2014) found that TPB was considerably less predictive of behavior when participants were not university students and when outcome measures were taken objectively rather than as a self-report [40]. This finding may provide partial explanation for low percentage of the variance in MVPA that TPB constructs accounted for in this study.

The results of the current study indicated that intention, rather than PBC, was significantly associated with MVPA. Children with greater intention to engage in MVPA reported more MVPA compared with children who expressed weaker intention. Consistent with TPB postulates, PBC, attitude, and subjective norms contributed minimally to account for additional variance beyond that explained by intention [24]. A similar conclusion was drawn in previous studies on PA among children [24, 28]. The finding that PBC was not significantly associated with behavior was surprising. According to Ajzen (1991), the strength of PBC in determining behavior depends on the accuracy with which PBC reflects actual control. However, the respondents in the present study, who are children aged 9 to 13 years, may not be accurate in judging the level of control they actually have over performing a behavior. Compared with adults, children differ in their concept of perceived control [41]. Furthermore, several external factors, such as influences exerted by their parents, can affect the control of children [42]. By contrast, the finding that intention instead of PBC is significantly related to MVPA among children contradicted the views of some researchers in China who overemphasized the negative association between external factors and PA participation among children, such as lack of space and facilities, extreme pressure to perform well in schools, and heavy home-work load, but disregarded the desire of children to engage in PA [43–45]. Although several external influences that control MVPA among Chinese children exist, igniting the desire of children to engage in PA may be more effective in improving their MVPA. Therefore, additional efforts may be necessary to stimulate the interest and desire of children to engage in PA. For example, making PA more “fun” and providing children with knowledge on the benefits of PA can increase their engagement in PA.

In terms of intention, the results of the current study indicated that attitude and PBC constructs were significantly correlated with PA intention. However, no significant relationship between social pressure from others and PA intention was found. The findings of this study are in line with those of other studies [20, 22, 28]. According to a review of the application of TPB to health-related behaviors, the subjective norm construct frequently did not achieve significance [46]. The insignificant relationship between social pressure from others and PA intention of children in this study corroborated this contention. Three reasons could explain this result. First, some participants in the present study were entering their teenage years, which were characterized by the development of adolescent autonomy (i.e., self-determination, decision making, and independence) [47]. Among early adolescents, autonomy may compel them to hold pressure from others (e.g., parents, teachers, and peers) in less regard than their personal volition [16]. Second, Chinese traditional culture is based on Confucian principles, which place “great emphasis on composed, reverential behavior that focused on a disposition of solemnity, self-control, and personal restraint of PA” [48] (p. 161) and successful scholarship [49]. Influenced by Confucian principles, other people (e.g., parents, teachers, and peers) highly value academic achievement at the “expense” of PA engagement [50]. The insignificant relationship between subjective norms and PA intention of children may be explained by the focus of other people on the academic achievement of children and the lack of care for their intention to engage in PA. Third, the insignificant relationship between subjective norms and PA intention may be related to the operationalization of the construct [46]. Subjective norms are only measured by a single item in the present study. Other normative components (e.g., descriptive and injunctive norms) may be encompassed by the standard measure [51]. This result suggests that future studies should consider other forms of social influences.

### 4.3. Gender Differences

Several studies reported that boys engage in PA at a higher rate than girls [12, 13, 29, 30]_._ For example, European girls spent significantly less time in MVPA compared with European boys [30]. In the US, girls aged 6 to 19 years spend less time in MVPA than their male counterparts [29]. The consistent conclusion is that Chinese boys are more active than girls [12, 13]. The current study confirmed gender differences in MVPA. Similarly, the finding also revealed that boys exhibited significantly higher intention to engage in PA than girls, which supported the results of other studies [24, 26]. However, according to Cohen (1992), the effect sizes of 0.20, 0.50, and 0.80 are small, medium, and large, respectively [52]. Despite two significant differences, their effect sizes (*η* ^2^ = 0.01 and *η* ^2^ = 0.02) did not satisfy the standard for even a small effect size, which indicated that the difference had low practical significance. No significant gender difference in the three TPB variables suggested that boys and girls had a similar attitude toward PA engagement and perceived similar social pressure and control on their PA engagement.

After comparing boys and girls in terms of TPB explanation, two important findings were established in the present study. First, TPB variables accounted for more variance in intention and MVPA behavior for boys than for girls. Hence, TPB performed better in the PA domain for boys than for girls. This finding explained the similarity of attitude, subjective norms, and PBC of boys and girls, whereas the PA intention and MVPA of boys were significantly higher than those of girls. However, the result is different from the findings of Rhodes et al. (2006), who have reported that TPB performs equally in explaining the activity intention and PA behavior of boys and girls [27]. These varying results may be related to cultural values. Many researchers have indicated that cultural values that tend to be endorsed by different populations moderate the effects among TPB constructs [22, 53]. In traditional Chinese culture, activeness, bravery, aggressiveness, and perseverance are valued by boys, whereas gentleness, kindness, and being approachable, sensitive, quiet, weak, and compliant are valued by girls [54]. Therefore, boys are supposed to play sports and stay active, but engaging in a similar behavior is unacceptable for girls. The Chinese culture may encourage boys to transform their positive attitude, pressure from others, and control on PA engagement into actual PA behavior more effectively. The second important finding of this study is that MVPA intention among boys is explained by their attitude and PBC, whereas the sole TPB variable significantly associated with intention among girls is PBC. This finding agrees with other studies, which have found that the variables significantly related to PA intention and behavior for boys and girls are different [23, 28]. Ajzen (1988) also acknowledged that gender could influence differences in the relative importance of TPB components that contributed to explaining behavioral intention [55]. A significant relationship exists between attitude and MVPA intention among boys but not among girls, which may be attributed to several factors, such as the lack of confidence of girls in sports competence [56, 57] and more opportunities for PA provided to boys than to girls [19]. Further research is necessary to explore which factor makes it difficult for girls to translate their positive attitude into high intention.

Gender differences found in MVPA duration, TPB variables, and the explanation capability of the TPB model may suggest that interventions tailored for different genders are necessary. In particular, additional efforts are required to encourage girls to engage in PA based on their lower MVPA level and intention. Control-based intervention seems to be warranted for all children, but more so even stronger for girls because it is the sole variable that is significantly related to intention among them. Apart from PBC, promotional efforts for boys should address their attitude toward PA engagement because of its significant association with intention.

## 5. Limitations and Future Research

The limitations of this research warrant further discussion. First, our inability to capture over 10% of the variance in MVPA suggests that additional critical determinants of MVPA have not been examined. Therefore, researchers should seek to investigate other factors that may contribute to explaining the PA behavior of Chinese children. These factors may include self-efficacy, moral norms, past PA behavior, self-identity, and environmental factors [39]. Another limitation was the lack of generalizability of the research results. The participants in this study are grades 3 to 5 students from Shanghai. Thus, the results may not be generalized to children of different ages and those coming from other areas in China. Future research with larger samples that cover more grade levels and other regions in China is recommended. Third, integrating TPB into other theories is necessary to provide a complementary explanation of TPB. For example, based on the possible influence of adolescent autonomy on TPB explanation of MVPA among children, future studies should be conducted to integrate TPB into self-determination theory to explain how people convert generalized motives into specific actions to engage in PA. Fourth, the present study revealed the contribution of TPB variables to MVPA but did not identify the specific factors related to subjective norms and PBC. Further studies that use qualitative methods (e.g., interviews and observation) are required to identify specific social pressure (e.g., pressure from parents, teachers, or peers) and perceived facilities and barriers in PA participation. These factors should be examined to determine how they influence the PA behavior of children.

## Figures and Tables

**Table 1:** Intercorrelations and descriptive data for theory planned behavior measures and physical activity behavior (*N* = 353).

Variable	Behavior	Intention	Attitude	Subjective norm	M	SD
Behavior					15.26	11.21
Intention	0.30^*∗∗*^				3.93	1.77
Attitude	0.07	0.33^*∗∗*^			4.99	1.76
Subjective norm	0.06	0.19^*∗∗*^	0.17^*∗∗*^		5.21	1.82
PBC	0.24^*∗∗*^	0.56^*∗∗*^	0.35^*∗∗*^	0.30^*∗∗*^	4.64	1.64

Note: ^*∗*^ *p* < 0.05; ^*∗∗*^ *p* < 0.01; ^*∗∗∗*^ *p* < 0.001.

**Table 2:** Gender differences among MVPA and TPB variables.

	Gender		
	Boys (*n* = 180)	Girls (*n* = 173)	*F*
Duration of MVPA	16.75 (12.53)	13.71 (9.43)	6.59^*∗*^
Behavioral intention	4.14 (1.84)	3.72 (1.69)	4.80^*∗*^
Attitude	4.99 (1.84)	4.98 (1.74)	0.01
Subjective norm	5.27 (2.63)	5.15 (1.80)	0.39
PBC	4.62 (1.75)	4.66 (1.52)	0.04

Note: ^*∗*^ *p* < 0.05; ^*∗∗*^ *p* < 0.01.

**Table 3:** Hierarchical multiple regression analysis predicting behavior.

Step	Variables	*R*	*R* ^2^	*R* ^2^ adj.	*B*	SE	*β*
1		0.301	0.090	0.088			
	Intention				1.896	0.321	0.301^*∗∗∗*^

2		0.097	0.097	0.092			
	Intention				1.551	0.388	0.246^*∗∗∗*^
	PBC				0.662	0.420	0.097

3		0.316	0.100	0.089			
	Intention				1.622	0.394	0.257^*∗∗∗*^
	PBC				0.784	0.442	0.115
	Attitude				−0.365	0.351	−0.057
	Subjective norm				−0.082	0.330	−0.013

4		0.334	0.112	0.099			
	Intention				1.495	0.396	0.237^*∗∗∗*^
	PBC				0.874	0.441	0.128
	Attitude				−0.351	0.349	−0.055
	Subjective norm				−0.107	0.329	−0.326
	Gender				−2.474	1.147	−0.110^*∗*^

Note: ^*∗*^ *p* < 0.05; ^*∗∗*^ *p* < 0.01; ^*∗∗∗*^ *p* < 0.001.

**Table 4:** Hierarchical multiple regression analysis predicting intention.

Step	Variables	*R*	*R* ^2^	*R* ^2^ adj.	*B*	SE	β
1		0.581	0.337	0.331			
	Attitude				0.145	0.047	0.144^*∗∗*^
	Subjective norm				0.017	0.045	0.017
	PBC				0.550	0.052	0.508^*∗∗∗*^

2		0.593	0.352	0.344			
	Attitude				0.144	0.047	0.143^*∗∗*^
	Subjective norm				0.544	0.052	0.511
	PBC				0.237	0.062	0.212^*∗∗∗*^
	Gender				−0.429	0.153	−0.121^*∗∗*^

Note: ^*∗*^ *p* < 0.05; ^*∗∗*^ *p* < 0.01; ^*∗∗∗*^ *p* < 0.001.

**Table 5:** Hierarchical multiple regression analysis predicting behavior by gender.

Step	Variables	Boys					Girls				
		*R* ^2^	*R* ^2^ adj.	*B*	SE	*β*	*R* ^2^	*R* ^2^ adj.	*B*	SE	*β*
1		0.09	0.09				0.07	0.07			
	Intention			1.70	0.39	0.31^*∗∗∗*^			1.84	0.49	0.27^*∗∗∗*^

2		0.11	0.09				0.08	0.07			
	Intention			1.36	0.47	0.25^*∗∗*^			1.41	0.62	0.21^*∗*^
	PBC			0.72	0.53	0.12			0.74	0.65	0.10

3		0.12	0.10				0.08	0.06			
	Intention			1.46	0.47	0.27^*∗∗*^			1.41	0.64	0.21^*∗*^
	PBC			0.86	0.53	0.14			0.79	0.71	0.11
	Attitude			−0.66	0.40	−0.12			−0.05	0.59	−0.01
	Subjective norm			−0.15	0.39	−0.03			−0.08	0.53	−0.01

Note: ^*∗*^ *p* < 0.05; ^*∗∗*^ *p* < 0.01; ^*∗∗∗*^ *p* < 0.001.

**Table 6:** Hierarchical multiple regression analysis predicting intention by gender.

Step	Variables	Boys					Girls				
		*R* ^2^	*R* ^2^ adj.	*B*	SE	β	*R* ^2^	*R* ^2^ adj.	*B*	SE	β
1		0.40	0.39				0.30	0.29			
	Attitude			0.22	0.07	0.21^*∗∗*^			0.08	0.06	0.08
	Subjective norm			−0.05	0.06	−0.05			0.09	0.06	0.09
	PBC			0.55	0.07	0.52^*∗∗∗*^			0.55	0.07	0.49^*∗∗∗*^

Note: ^*∗*^ *p* < 0.05; ^*∗∗*^ *p* < 0.01; ^*∗∗∗*^ *p* < 0.001.
